# Treatment of distal radial fractures with antegrade intra-medullary Kirschner wires

**DOI:** 10.1007/s11751-013-0161-z

**Published:** 2013-06-06

**Authors:** Mohamed F. Mostafa

**Affiliations:** Department of Orthopedic Surgery and Traumatology, Faculty of Medicine, Mansoura University, PO Box 2, Mansoura, 35516 Egypt

**Keywords:** Distal, Radial, Colles, Antegrade, K-wire

## Abstract

The treatment of unstable Colles-type distal radial fractures remains a challenge. A prospective study was conducted to evaluate the outcomes of the treatment of unstable distal radial fractures using antegrade intra-medullary K-wires. Twenty-eight Colles-type distal radial fractures were selected excluding comminuted intra-articular and Barton’s fractures. The blunt tips of intra-medullary K-wires were introduced in an antegrade direction to support the subchondral bone of the distal fragment. The scoring system of Green and O’Brien modified by Cooney et al. was used for the final clinical evaluation. The radiological outcomes were evaluated using the scale proposed by Stewart et al. After a mean follow-up of 34 months (range 14–46), 17 patients were rated clinically excellent, seven good, three fair and one poor. The mean loss of radial height, radial inclination, volar tilt and ulnar variance was 0.9 mm, 1.9°, 0.5° and 0.4 mm, respectively. These results were comparable with the values reported in other pinning studies. Only one patient complained of skin irritation and painful bursitis in the forearm; otherwise, no complications related to tendon or nerve injury were encountered. One patient had protrusion of K-wire into the wrist joint. The technique proved to be effective in maintaining reduction in distal radial fracture with low rate of soft tissue complications.

## Introduction

Fractures of the distal radius are common injuries accounting for one-sixth of all fractures seen in the emergency room [1–3]. Colles [4] initially stated that the wrist eventually gains freedom in all of its motions and be completely exempt from pain. This perpetuated the concept that distal radial fractures could be treated non-operatively with an expected good functional outcome especially in the elderly. However, more than half of these fractures involve either the distal radioulnar or the radiocarpal joints and, although initially reducible by manipulation, may be inherently unstable and collapse with simple cast immobilization. Recent reports have confirmed a direct correlation between late functional results and residual deformity. Therefore, the trend has shifted towards restoring articular congruency and bony anatomy of the distal radius using operative means [5–8].

Percutaneous pinning is a simple, minimally invasive technique to maintain the reduction in and prevent redisplacement of the fragments [9, 10]. Retrograde Kirschner-wire (K-wire) fixation can be extrafocal-bicortical [9, 11] or intra-focal-unicortical [12]. Many complications have been reported after retrograde pinning for distal radial fractures, including soft tissue irritation, injury to radial sensory nerve and extensor tendons, pain, algodystrophy, pin tract infection especially when left outside the skin and loss of reduction [13–16].

Sato et al. [17] introduced a technique of fixation for the unstable extra-articular distal radial fractures by manual reduction and antegrade intra-medullary pinning. They found this procedure beneficial in controlling the distal radial angulation with less soft tissue complications. The current prospective study was conducted to evaluate the clinical and radiological outcomes of management of unstable distal radial fractures in adults by closed manipulation and antegrade intra-medullary K-wire fixation after some modifications to the original technique.

## Patients and methods

From February 2007 to May 2011, 28 patients with unstable Colles-type distal radial fractures were selected and managed by closed reduction and percutaneous antegrade intra-medullary K-wire fixation. The following criteria of fracture instability proposed by Lafontaine et al. [18] were used for selecting the patients: dorsal angulation of 20° or more, dorsal comminution, intra-articular fracture, combined ulnar fracture, radial shortening of 5 mm or more and age over 60 years. Fractures in skeletally immature patients, severely comminuted intra-articular fractures and Barton’s fractures were excluded from this study. All patients were briefed about the technique and its possible complications, and an informed consent was obtained to participate in the study.

There were 17 men and 11 women with an average age of 43 years (range 22–75) at the time of presentation. The left side was involved in 19 patients. Thirteen fractures were caused by an accidental fall, 10 were caused by road traffic accidents, and five were sports injuries. The fracture was open Gustilo and Anderson grade I in two cases and was associated with posterior elbow dislocation in one case, comminuted supracondylar fracture of the femur in one, trochanteric fracture in one and visceral injury in one. According to Frykman’s criteria [19], 19 fractures were classified as type I and II, three as type III and IV and six as type V and VI. According to the AO/Orthopedic Trauma Association criteria [20], fractures were graded A2-2 in six patients, A3-2 in 19 and C1-2 in three.

### Surgical procedure

**Fig. 1:**
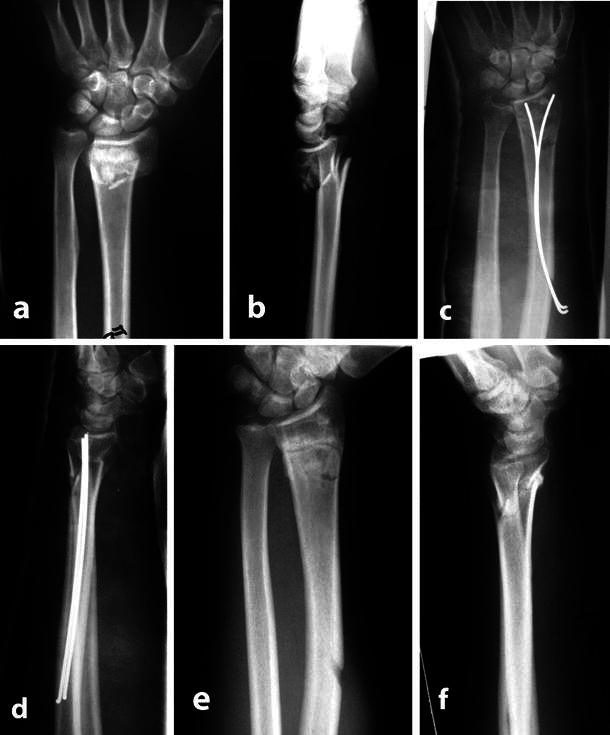
45-year-old male with (**a** and **b**) Frykman’s type II (AO: A 3-2) fracture of distal-end left radius; (**c** and **d**) post-operative radiographs showing the blunt ends of K-wires supporting the subchondral bone; (**e** and **f**) radiographs after removal of K-wires

**Fig. 2:**
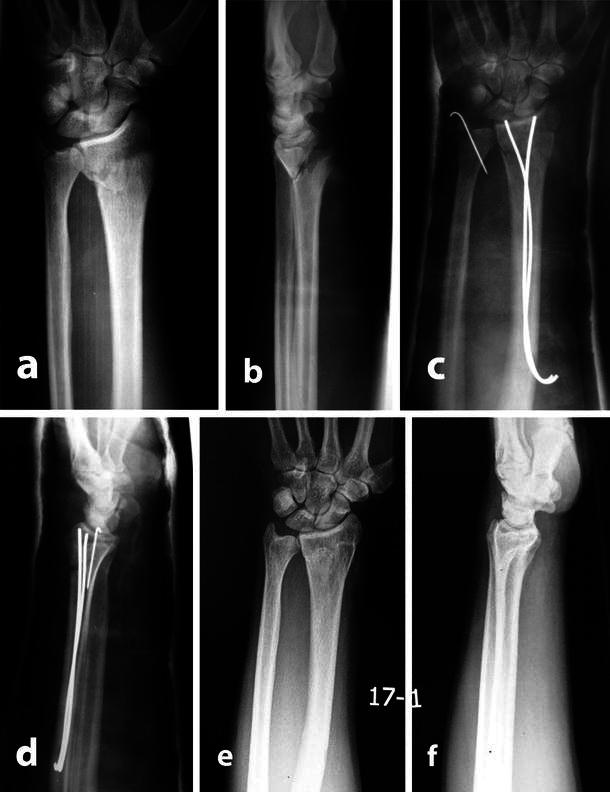
**a** and **b** preoperative radiographs of a 37-year-old male with Frykman’s type II (AO; A 2-2) fracture of left radius and fracture of base ulnar styloid; (**c** and **d**) post-operative radiographs showing retrograde K-wire used for fixation of ulnar styloid; (**e** and **f**) radiographs one and a half years after removal of K-wires showing an excellent radiological score

The surgery was performed under general anaesthesia in 11 patients and under Bier’s intravenous block anaesthesia in 17. The average time from injury to surgery was 2.2 days (range 1–9). Finger-trap traction was not used, and the patient’s forearm was maintained parallel to the ground on a radiolucent side arm. After routine sterilization and draping, a 3-cm longitudinal skin incision was made on the dorsoradial aspect of the mid-radius about 10–12 cm proximal to the radial styloid. The interval between the extensor carpi radialis brevis muscle and the extensor digitorum communis muscle proximal to the abductor pollicis longus muscle was developed. Then, the cortex of the radius dorsal to the pronator muscle insertion was exposed. One slanting hole was made with a 3.5-mm drill bit in large-size bones, provided that the diameter of the drill hole is less than half the width of bone at the level of drilling. In patients with small-size bones, two drill holes at two different levels were made with 2-mm drill bit to avoid the stress riser effect. At first, the drill bit was directed perpendicular to the bone and then obliquely at an angle of 45°–60° with care to avoid penetration of the far cortex. A drill sleeve was used to protect the soft tissues. Through these holes, two 1.5-mm K-wires were inserted manually into the medullary canal over a T-handled drill chuck. The tip and body of the K-wires were slightly prebent by pliers. During insertion, the prebent wires formed a smooth curve in the medullary canal. At first, the tips of the wires were directed radially and once reached the fracture site; the distal fragment was manipulated while traction was applied in the axis of the second and third digits and counter-traction on the upper arm. The quality of the reduction was then checked with fluoroscopy in anteroposterior and lateral projections by rotating the C-arm around the wrist while the patient’s hand was held steadily. In three patients, there was a non-displaced or minimally displaced intra-articular fracture, and a transverse K-wire was used for fixation. One intra-medullary K-wire was rotated 180° and pushed towards the ulnar side of the distal fragment, and the other wire was directed to the radial styloid (Fig. 1). The blunt tips of the K-wires were used intra-medullary to avoid the inadvertent protrusion into the wrist joint by the sharp tips. K-wires were advanced till rested against the subchondral bone. The proximal ends of the K-wires were bent and buried in the subcutaneous tissues. In nine patients, ulnar styloid was fractured through the base and displaced and therefore was fixed with a retrograde percutaneous K-wire (Fig. 2). After surgery, a short-arm cast was applied for 3 weeks, leaving fingers and elbow motions unrestricted. In the patient with posterior elbow dislocation, a long-arm cast was applied till the soft tissue healed. The wrist was supported with a short volar splint for an additional 3 weeks. Then, patients were asked to move their wrist and forearm and to strengthen their muscles by clenching a soft ball firmly. K-wires were removed after an average time of 8.9 weeks (range 8–11) from surgery under local anaesthesia in the operating room.

All the patients were evaluated clinically for pain, functional status, range of motion and grip strength after a follow-up with an average duration of 34 months (range 14–46). Range of motions and grip strength of the affected wrists were measured and compared with those of the contralateral healthy wrists. The scoring system of Green and O’Brien [21] modified by Cooney et al. [22] (Table 1) was used for the final clinical assessment of patients. During the period of fixation with K-wires, patients were monitored for the possible complications, such as skin irritation by wires, paraesthesia of the sensory branches of the radial nerve, injury to the extensor tendons, reflex sympathetic dystrophy (RSD) and carpal tunnel syndrome. Radiological evaluation included the measurement of radial height, radial tilt, dorsal to volar tilt and ulnar variance on anteroposterior and lateral radiographs before surgery, immediately after surgery and at the final follow-up. These measures were used for the radiological evaluation of patients based on the radiological scoring system proposed by Stewart et al. [23] (Table 2). Follow-up radiographs were also checked for any evidence of ligament injury (scapholunate, lunotriquetral, DISI and VISI) or complications related to K-wires, such as breakage, migration, backing-out and protrusion into the wrist joint.

**Table 1:** Clinical scoring system of Green and O’Brien modified by Cooney

Items	Findings	Score (points)
Pain	None	25
	Mild, occasional	20
	Moderate, tolerable	15
	Severe or intolerable	0
Functional status	Returned to regular employment	25
	Restricted employment	20
	Able to work but unemployed	15
	Unable to work because of pain	0
Range of motion	Full	25
	75–99 % of normal	15
	50–74 % of normal	10
	25–49 % of normal	5
	Less than 25 % of normal	0
	Or evaluating dorsiflexion-palmar flexion arc of injured hand	
	120° or more	25
	91–119°	15
	61–90°	10
	31–60°	5
	30° or less	0
Grip strength	Normal	25
	75–99 % of normal	15
	50–74 % of normal	10
	25–49 % of normal	5
	0–24 % of normal	0

Results: excellent 90–100, good 80–89, fair 65–79, poor <65

**Table 2:** Radiological and anatomical scoring system

Final dorsal tilt (°)	Neutral/volar	1–10	11–14	15+
Loss of ulnar variance (mm)	0–3	4–6	7–11	12+
Loss of radial inclination (°)	0–4	5–9	10–14	15+
Score for each	0	1	2	4

Grades: 0 excellent, 1–3 good, 4–6 fair, 7–12 poor

The results were tabulated and statistically analysed using the statistical package for social sciences version 17.0 (SPSS Inc, Chicago, IL, USA). Pearson’s chi-square test and one-way ANOVA test were used to define relations between clinical, radiological and end results. A *p* value of <0.05 was considered significant.

## Results

According to Cooney’s modification of Green and O’Brien’s score for clinical outcome, 17 (61 %) patients were graded excellent, seven (25 %) good, three (11 %) fair and one (4 %) poor. This clinical score was not statistically related to the type of fracture. However, AO type A fractures showed the highest clinical score compared with type C fractures. The poor result was related to the development of RSD, pain and arthrofibrosis with marked limitation of wrist movement. The patient was reluctant to move after removal of the splint. Among the patients with fair clinical results, one had RSD that responds partially to physiotherapy, one had other associated fractures that interfered with rehabilitation after removal of the cast, and one had pain at the inferior radioulnar joint. Age of patients appeared as an important factor for clinical prognosis. The excellent and good results were achieved more in younger patients than fair and poor results (*p* = 0.004). No cases of tendon injury, vascular complication, superficial or deep infection were encountered. Irritation of the sensory branches of the radial nerve caused by the retrograde K-wire was seen in one patient (AO type C) and resolved completely few weeks after the K-wire removal. However, no cases of median nerve injury or compression were encountered. Skin irritation and painful bursitis at the buried ends of antegrade K-wires occurred in one patient and resolved spontaneously after planned removal of the K-wires. Reflex sympathetic dystrophy developed in two patients and mostly due to the early refusal to mobilize fingers rather than to the prolonged immobilization. The condition was recovered with physiotherapy in one patient followed by arthrofibrosis and marked limitation of wrist movement in the other.

All fractures healed uneventfully after an average time of 7.7 weeks (range 6–9). The mean time of radiological healing was significantly shorter in less comminuted (type I and A2-2) fractures than in more comminuted (type VI, A3-2 and C1-2) fractures (*p* = 0.01 and 0.02, respectively). The mean radial height improved from 2.4 mm (range 0–11) preoperatively to 12 mm (range 8–13) post-operatively and reduced to 11 mm (range 5–13) at the final follow-up with a mean loss of reduction of 0.9 mm (range 0–7). The mean radial inclination varied from 7.1° (range 3°–16°) preoperatively to 21.5° (range 16°–27°) post-operatively and was 19.6° (range 10°–27°) at the final follow-up, giving a mean loss of reduction of 1.9° (range 0°–11°). The mean loss of radial height and radial inclination was significantly correlated to the final clinical outcome (*p* = 0.02). The mean distal radial tilt changed from 17.1° dorsal tilt preoperatively to 7.9° volar tilt post-operatively and averaged 7° volar tilt at the final follow-up evaluation. There was no statistical significance between the changes in radial tilt and the final clinical outcome. The ulnar variance was positive in 25 patients and neutral in three preoperatively with a mean of 2.5 mm (range 0–8 mm). After surgery, ulnar variance changed to negative in four patients (mean 1 mm) and remained positive in six (mean 1.3 mm). At the final follow-up, ulnar variance continued to be negative in four patients and positive in nine (mean 1.9 mm) with a mean loss of reduction of 0.39 mm (range 0–3 mm). The positive ulnar variance at the final follow-up was significantly related to fair and poor clinical outcomes (*p* = 0.01). Twenty-three patients were rated excellent and five good according to the anatomical scale of Stewart [23]. A statistically significant correlation was detected between high radiological score and good clinical outcome (*p* = 0.003).

No evidence of wrist arthrosis or step-off of the radial articular surface was detected. There were two cases of VISI and one of DISI at the final follow-up. No cases of wire breakage or backing-out were encountered. Protrusion of K-wire into the radiocarpal joint occurred in one patient, a “75-year-old woman” who had osteoporosis and inadvertent penetration of the radial articular surface during wire insertion. However, no bone erosion of carpal bones could be detected radiographically till the removal of K-wires.

## Discussion

Conservative treatment of minimally displaced and stable fractures of the distal end of the radius usually shows a good outcome; however, the ideal treatment of severely displaced and unstable fractures remains controversial. Percutaneous pinning, pin and plaster, external fixation and plating have been reported to reduce the risk of malunion of distal radial fracture and the subsequent mid-carpal instability, osteoarthritis and pain [24]. Percutaneous pinning such as trans-styloid and Kapandji procedure is a simple and cost-effective technique provided that the distal radius is not severely comminuted or osteoporotic [3]. Because the pin is inserted close to the wrist joint, complications such as skin irritation, sensory nerve injury, tendon rupture and RSD are common [13–16]. To avoid these complications and to stabilize distal radial fractures, antegrade intra-medullary K-wire fixation was performed. The reported complication rate of pin and plaster was 42 % [25], trans-styloid pinning 10 % [26], Kapandji procedure 20 % [26] and trans-styloid pinning with bone grafting 33 % [27]. In the current study, the soft tissue complication rate related to the antegrade wire was 4 %, which is apparently lower than that of other studies using retrograde pinning.

Hochwald et al. [28] found that even open pinning caused tendon and nerve injury around the wrist joint in 15 % of the cases. Even if tendons and nerves escape injury, skin irritation around the pins occasionally prohibits patients from moving the fingers and wrist. Irritation of the radial sensory nerve was observed in only one patient and was related to the use of a retrograde transverse K-wire for stabilization of dorsoulnar fragment. Because the proximal ends of the pins were buried in the subcutaneous tissues of the forearm, irritation of skin was minimal during finger and wrist movement. Skin irritation and painful bursitis were seen in one patient in whom K-wires were not buried enough and resolved spontaneously after the planned removal of the K-wires. The site of pin insertion was consistent with that described by Melone [29] and used by Sato et al. [17]. It was located on the dorsoradial cortex of the radial diaphysis between the extensor carpi radialis brevis and the extensor digitorum communis where sensory and motor branches of the radial nerve were absent. This could explain the absence of tendon injury or attrition and delayed tendon rupture.

**Fig. 3:**
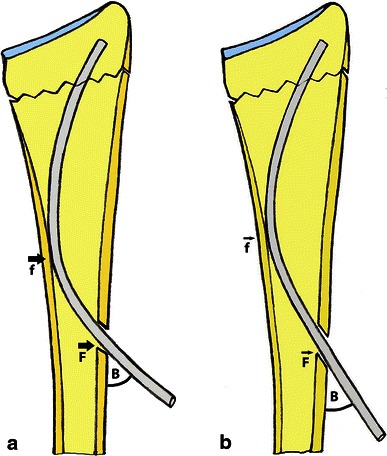
Diagram showing the relationship between the inclination angle of the entry hole to the cortex and the friction force. **a** When the entry hole inclination angle “*B*” is almost 40°–60° to the humeral cortex, the friction forces at the entry hole “*F*” and at the *inner wall* of the contralateral cortex “*f*” increased, and this may prevent K-wire from backing-out; **b** when the entry hole inclination angle “*B*” is 30° or less, the friction forces “*F*” and “*f*” decreased and the K-wire may easily back out

The mean radial shortening after trans-styloid pinning was reported to be 1 mm [2], whereas that after Kapandji procedure was 2 mm [26] and trans-styloid pinning associated with bone grafting 0 mm [27]. Sato et al. [17], in their study of using antegrade intra-medullary K-wire for fixation of distal radial fracture, reported a radial shortening value of 2.6 mm greater than the values in other studies. They explained this by the protrusion of the sharp tips of K-wires into the subchondral bone with subsequent collapse at the fracture. Therefore, they recommended the supplement with external fixation or trans-styloid pinning with or without bone grafting in cases with major shortening of more than 5 mm. In the present study, the mean radial shortening value of 0.96 mm was considered less than that reported by Sato et al. [17]. This suggests that antegrade intra-medullary K-wires were effective to maintain the radial height provided that the blunt tips of the wires were used to support the subchondral bone and the inadvertent penetration of the articular surface was avoided. Also, the increased friction force between K-wires and bone cortex by increasing the inclination angle of the entry hole could prevent the wires from backing-out and collapse at the fracture (Fig. 3).

The reported volar tilt at the final follow-up was 0°–2° for pin and plaster [25], 3° for trans-styloid pinning [26], 3° for Kapandji procedure [26], 2° for trans-styloid pinning with bone grafting [27] while averaged 5° for antegrade intra-medullary K-wire fixation [17]. The mean volar tilt of 7° observed in the present study was comparable with that of other studies and indicated that this technique could prevent redisplacement in dorsal tilt. Several authors noted a close relationship between anatomic reduction in the displaced distal radial fracture and good functional outcome [5–8]. In the current study, a significant correlation was detected between the radiological score and the final clinical outcome with fair and poor clinical results related to radial shortening, loss of radial inclination and positive ulnar variance but not to loss of volar tilt. In agreement with Rosati et al. [30], 0° volar tilt did not impair the range of motion of the wrist because it could be compensated for by the mid-carpal joint. However, prognostic evaluation based solely on anatomical background seems difficult, as results also depend on other important factors such as age, metabolic bone condition, other associated injuries, quality of rehabilitation and occurrence of complications, such as reflex sympathetic dystrophy and subsequent joint stiffness. It also appears that a more comminuted fracture makes prognosis worse, as pointed out by Trumble et al. [31].

## Conclusions

Despite the relatively small number of cases, the results were encouraging and proved that antegrade intra-medullary K-wire fixation is an effective technique for stabilization and prevention of secondary displacement of distal radial fractures. Care must be taken in selecting patients as this technique is not suitable for fractures with marked intra-articular or metaphyseal comminution. This study gives the necessity to plan a biomechanical study to evaluate the efficacy of intra-medullary K-wire in withstanding axial, shearing and bending forces.
